# Psychological Distress Indicators Among Pregnant Women Reporting Intimate Partner Violence in Primary Care: Implications for Trauma-Informed Antenatal Care in Nigeria

**DOI:** 10.3390/healthcare14203508

**Published:** 2026-10-19

**Authors:** Gabriel Dogbanya, Mona Mittal

**Affiliations:** Department of Family Science, School of Public Health, University of Maryland, College Park, MD 20742, USA

**Keywords:** intimate partner violence, psychological distress, primary care, pregnancy, Nigeria

## Abstract

**Background**: Intimate partner violence (IPV) during pregnancy is a significant public health concern in low-resource settings. Primary care antenatal clinics are often the first point of contact for pregnant women and provide important opportunities to identify both violence and related psychological distress. This study examined the prevalence of IPV during pregnancy, the reporting of psychological distress indicators (low self-esteem, sleep disturbance, and suicidal ideation) among women reporting IPV, and the association between IPV and timing of antenatal care (ANC) initiation in a low-resource primary care setting in Nigeria. **Methods**: We analyzed cross-sectional data from 463 pregnant women receiving routine antenatal care at a primary health center in Abuja, Nigeria (February–July 2024). IPV during the index pregnancy was assessed via an interviewer-administered survey. Psychological distress indicators were measured using single-item self-report questions assessing perceived self-esteem, sleep disturbance, and suicidal thoughts attributed to violence. Multivariable logistic regression models examined associations between IPV and late ANC initiation (>12 weeks’ gestation), adjusting for sociodemographic and partner-related characteristics. Additional analyses examined the associations between late ANC initiation and perceived psychological distress indicators among women reporting IPV. **Results**: IPV during the index pregnancy was reported by 68.7% of participants. Among women reporting IPV, high proportions also reported psychological distress indicators, including low self-esteem (77.1%), sleep disturbance (67.3%), and suicidal thoughts (approximately one-third). Women reporting IPV had significantly lower odds of delayed ANC initiation (aOR = 0.37; 95% CI: 0.19–0.69). Individual psychological indicators were not independently associated with ANC timing after adjustment. **Conclusions**: Although women reporting IPV were more likely to initiate ANC early, they frequently also reported psychological distress indicators, highlighting routine ANC visits as important opportunities for trauma-informed identification, assessment, and referral.

## 1. Introduction

Intimate partner violence (IPV) during pregnancy is a major public health and human rights concern that affects women across diverse sociocultural contexts [1]. IPV during pregnancy not only threatens women’s physical safety but also increases the risk of psychological distress, postnatal depression, perinatal mortality, low birth weight, and preterm delivery [2,3]. The burden of IPV during pregnancy is particularly pronounced in Nigeria, where studies have reported prevalence estimates ranging from 33% to 67%, underscoring the persistence of this maternal health challenge [4,5]. In low-resource primary care settings such as Nigeria, primary healthcare facilities often represent the first, and sometimes only, point of contact for pregnant women. Consequently, antenatal care provides an important opportunity not only to address obstetric needs but also to identify women experiencing IPV and associated consequences.

An expanding body of literature demonstrates that women exposed to IPV during pregnancy are more likely to report a range of psychological challenges, such as suicidal ideation, sleep disturbance, and diminished self-esteem [6,7,8,9,10,11]. A scoping review of 12 studies identified IPV exposure as a significant predictor of poor sleep outcomes [12]. Consistent with these findings, other studies have reported that IPV-related stress is associated with increased sleep disturbance and poor sleep quality, particularly during pregnancy [13,14]. Low self-esteem has also emerged as both a consequence of IPV and a potential effect modifier of its effects on mental health. Beyond the context of IPV, diminished self-esteem is independently associated with increased risks of depression, anxiety, and broader psychological morbidity [15,16,17,18,19,20]. Evidence further suggests that low self-esteem may exacerbate the adverse mental health effects of IPV, including suicidal ideation, whereas higher self-esteem may serve as a protective buffer [20,21,22]. Consistent with these findings, women exposed to IPV during pregnancy have been shown to have elevated odds of suicidal ideation compared with those who are not exposed [3]. Collectively, these findings suggest that IPV during pregnancy is associated with substantial psychological vulnerability, highlighting antenatal care as a critical opportunity for identifying women who may benefit from trauma-informed assessment, referral, and support.

Although these psychological effects are well documented, considerably less is known about how they present among pregnant women reporting IPV within routine primary care settings, particularly in low-resource countries. Understanding these experiences is clinically important because antenatal care often provides the first opportunity to identify women experiencing IPV and psychological vulnerability. Some studies suggest that pregnant women experiencing psychological distress are more likely to miss prenatal care milestones or delay care [23,24], whereas others have reported earlier initiation of prenatal care among women exposed to IPV [25]. Further research is therefore needed to better understand psychological distress and antenatal care engagement among women reporting IPV during pregnancy in primary care settings. Psychological distress refers to a state of emotional suffering characterized by symptoms of depression (e.g., loss of interest, suicidal ideation, low self-esteem, and hopelessness) and anxiety (e.g., restlessness and tension) and may also manifest through somatic symptoms such as insomnia, headache, and low energy [26,27]. Although psychological distress is widely used as an indicator of mental health, its conceptualization varies across studies, with no consensus criteria defining it [28]. In this study, psychological distress is conceptualized as encompassing perceived low self-esteem, poor disturbance, and suicidal thoughts.

Primary care settings represent a critical entry point for identifying pregnant women experiencing IPV and associated psychological distress while providing an important opportunity to deliver trauma-informed care to women with trauma exposure. Trauma-informed care emphasizes understanding how trauma can affect individuals and integrating this knowledge into all aspects of service delivery, including adapting care practices to promote safety, trust, and responsiveness to the needs of trauma-affected individuals [29]. These principles are being advocated in maternity care [30,31]. The World Health Organization endorses healthcare-based IPV screening and response as an essential strategy for early IPV identification and support [32]. In Nigeria, primary health centers form the foundation of antenatal care services for many women, particularly those living in resource-constrained communities [33,34]. As a result, antenatal care (ANC), a core component of primary healthcare, provides a valuable opportunity to identify women experiencing IPV and psychological distress and facilitate timely interventions [23,35]. Identifying pregnant women experiencing IPV constitutes the first and most critical step toward ensuring safety [36,37]. Also, a better understanding of the psychological distress experienced by women dealing with IPV while they are engaged in ANC may help inform more effective trauma-informed screening, referral, and support strategies within primary healthcare settings [38].

The Stress Process Model and the Ecological Model for Violence Against Women provide complementary perspectives for understanding IPV during pregnancy [39,40]. The Stress Process Model conceptualizes IPV as a chronic interpersonal stressor that may undermine emotional security, disrupt sleep regulation, erode self-worth, and increase vulnerability to suicidal ideation during pregnancy. The Ecological Model, as adapted by Heise into the Ecological Framework for Violence Against Women, emphasizes that women’s experiences of IPV and access to support are shaped by interacting influences operating at individual, relationship, community, systems, and societal levels [41]. Together, these frameworks highlight the importance of primary healthcare settings as critical contexts for identifying women experiencing IPV and associated psychological distress and facilitating appropriate support and referral.

### Current Study

Although research has demonstrated associations between IPV during pregnancy and adverse psychological outcomes, relatively little is known about psychological distress among IPV-exposed women who are already engaged in routine ANC, particularly within primary healthcare settings in low- and middle-income countries where both IPV prevalence and maternal health burdens are disproportionately high [42]. This represents an important gap because ANC encounters provide opportunities not only to identify IPV but also to recognize psychological distress and initiate appropriate support. Better understanding these experiences may help inform trauma-informed maternal healthcare strategies that integrate IPV screening with psychological assessment and referral.

Guided by the Stress Process Model and the Ecological Model, this study examined psychological distress among pregnant women reporting IPV in a primary care antenatal clinic in Nigeria. Specifically, the study aimed to (1) estimate the prevalence of IPV during pregnancy among women receiving ANC in a primary care setting, (2) describe psychological distress indicators among women reporting IPV, and (3) examine the associations of IPV and psychological distress indicators with timing of ANC initiation. By addressing these aims, this study seeks to inform trauma-informed primary healthcare approaches that strengthen identification of IPV and psychological distress during routine ANC, particularly in resource-constrained primary care settings.

## 2. Methods

### 2.1. Data Source and Study Population

This study utilized secondary data obtained from a cross-sectional survey of pregnant women receiving care at a maternal and child primary care center in Kado Kuchi, Abuja Municipal Council, Abuja, North-Central Nigeria, between February 2024 and July 2024. The data were collected among consenting women using a convenience sampling approach, with a total of 480 respondents during the study period. Eligible participants were pregnant women in their first to third trimester, aged 18 years and above, who provided informed consent to participate and were on a routine clinic visit. Women who came for an emergency visit, were in labor and in any clinical distress, or those who refused consent were excluded from the study. The instrument for data collection was a well-structured, pretested, interviewer-administered survey questionnaire administered by trained research assistants (nurses) in a private setting to ensure confidentiality. Women who reported severe IPV symptoms or psychological distress, including suicidal ideation, were referred for further assessment and appropriate management. The details and outcomes of these referrals are beyond the scope of this study. Ethical approval for the study was obtained from the Health Research and Ethics Committee (HREC) of Ahmadu Bello University Teaching Hospital, Zaria, Nigeria.

### 2.2. Measures

Exposure: Intimate Partner Violence (IPV): Intimate partner violence was assessed by asking respondents if they had experienced abuse by their husband/partner in the index pregnancy. The response was binary (Yes/No).

### 2.3. Outcomes

Psychological Distress: Self-reported psychological distress was assessed by responses to single-item questions on whether the IPV experienced in the index pregnancy led them to experience feelings of low self-esteem and sleep deprivation and made them feel suicidal (suicidal ideation). The responses were coded dichotomously for all 3 psychological distress indicators.

Timing of antenatal engagement: There are two outcome categories: ≤12 weeks and >12 weeks. Those who booked care in the first 12 weeks were categorized as booked early, while those who booked after 12 weeks were categorized as booked late.

### 2.4. Covariates

Covariates for the study included parity, number of children alive, maternal education (primary, secondary, and tertiary), maternal occupation (farmer, artisan, or civil servant); partner’s education (primary, secondary, and tertiary) & occupation (farmer, artisan, or civil servant); partner job insecurity (binary: yes/no); partner substance abuse (binary: yes/no); and low social support (binary: yes/no). These variables were controlled for in the statistical analysis.

### 2.5. Analysis

All questionnaires were examined for completeness and internal consistency. Seventeen cases with missing or inconsistent information were removed, resulting in a final analytic sample of 463 participants. All analyses were conducted using Stata version 17. Statistical significance was determined at α = 0.05, and effect estimates are presented as odds ratios (ORs) with 95% confidence intervals (CIs). Descriptive and bivariate analyses were conducted to examine the association between IPV exposure during pregnancy and participants’ sociodemographic characteristics. The prevalence of self-reported psychological symptoms among IPV-exposed women was summarized using frequencies and percentages. A binary logistic regression, adjusting for sociodemographic and partner characteristics, was used to examine the association between IPV exposure and late ANC initiation (model 1) and the association between psychological distress and ANC timing among IPV-exposed women (model 2). Analyses examining psychological distress indicators were restricted to women reporting IPV because the psychological questions specifically referred to distress attributed to violence.

## 3. Results

A total of 463 women were included in the analysis, of whom 318 (68.7%) reported experiencing IPV during the index pregnancy. Table 1 summarizes the sociodemographic and reproductive characteristics of participants by IPV exposure status. The mean age of participants was 31.5 ± 7.6 years. Maternal age was similar between women exposed and not exposed to IPV, with no statistically significant difference observed. However, women who experienced IPV had a significantly higher number of living children and higher parity compared with those who did not experience IPV. Maternal education was inversely related to IPV exposure; women with only primary education had higher IPV exposure compared to those with tertiary education. Maternal occupation also differed significantly by IPV exposure. IPV exposure was more common among farmers and civil servants, whereas unexposed women were more likely to be artisans. A strong, statistically significant association was observed between IPV exposure and the timing of antenatal care (ANC) initiation (*p* < 0.001). Women exposed to IPV were more likely to initiate ANC early (≤12 weeks’ gestation) compared with unexposed women (48.7% vs. 26.2%). Conversely, late booking (>12 weeks) was more common among women without IPV exposure (Table 1). Among IPV-exposed women, low self-esteem was the most prevalent self-reported psychological indicator, reported by 77.1% of participants. Sleep disturbance was also highly prevalent, affecting 67.3% of IPV-exposed women. Suicidal ideation was reported by nearly one-third of IPV-exposed women (Figure 1).

Table 2 presents the results of a multivariable logistic regression model examining the association between IPV exposure during the index pregnancy and delayed initiation of antenatal care (ANC > 12 weeks’ gestation). After adjusting for maternal sociodemographic characteristics, reproductive history, partner-related factors, and social support, IPV exposure was significantly associated with lower odds of delayed ANC initiation. Women who experienced IPV during pregnancy had 63% lower odds of initiating ANC late compared with women who were not exposed to IPV (aOR 0.37, 95% CI: 0.19–0.69; *p* = 0.002). This association is consistent with the unadjusted analysis (OR 0.37, 95% CI: 0.24–0.57; *p* < 0.001).

Maternal age, parity, number of living children, and occupation were not independently associated with late ANC booking. Unexpectedly, maternal education was significantly associated with delayed ANC initiation. Compared with women with primary education, those with secondary education had more than twice the odds of initiating ANC after 12 weeks’ gestation (aOR 2.10, 95% CI: 1.08–4.08; *p* = 0.028). Women with tertiary education had 2.5 times the odds of delayed ANC initiation compared with women with primary education (aOR 2.52, 95% CI: 1.39–4.55; *p* = 0.002). Partner education, job insecurity, and substance use were not significantly associated with ANC timing. However, partner occupation showed a strong protective association. Having a partner who was a farmer (aOR 0.22, 95% CI: 0.09–0.51; *p* < 0.001) or an artisan (aOR 0.31, 95% CI: 0.19–0.50; *p* < 0.001) was associated with significantly lower odds of delayed ANC initiation compared with partners who were civil servants. Low social support was not associated with delayed ANC initiation (aOR 0.68, 95% CI 0.33–1.42; *p* = 0.303).

Table 3 presents the results of multivariable logistic regression analyses examining the association between individual self-reported psychological distress indicators and delayed ANC initiation among women reporting IPV. None of the individual self-reported psychological distress indicators, self-esteem, sleep disturbance, or suicidal ideation, was significantly associated with delayed ANC initiation after adjustment for maternal sociodemographic, reproductive, partner-related, and social support characteristics. Maternal age, parity, number of living children, maternal occupation, and social support were not significantly associated with ANC timing across all models. Maternal education was associated with delayed ANC initiation across all models. Compared with women with primary education, those with secondary education had more than three times the odds of initiating ANC after 12 weeks’ gestation (aOR ≈ 3.6; *p* = 0.003 across models). Women with tertiary education also had significantly higher odds of delayed ANC initiation compared to women with primary education (aOR ≈ 3.4; *p* = 0.001). Partner characteristics were generally not associated with ANC timing; however, having an artisan partner was significantly associated with ANC timing across all models (*p* < 0.001).

## 4. Discussion

This study adds to the growing evidence on psychological distress experienced by IPV-exposed women during pregnancy and its implications for antenatal engagement in low-resource settings. Our study found that in this low-resource primary care setting, women reporting IPV were already accessing antenatal care (ANC), with a substantial burden of psychological distress. This suggests that routine ANC encounters represent critical opportunities for early identification of IPV, assessment of psychological well-being, and linkage to trauma-informed support services.

The prevalence of IPV in pregnancy in our study setting was 68.7%. This is consistent with the reports by Kana et al. [5], who found the prevalence of IPV in pregnancy to be 67% in Kaduna State, Nigeria. However, this is considerably higher than the IPV prevalence (33%) reported by Oche in Sokoto State, Nigeria [4]. This difference may be attributed to variation in study settings. Unlike the Sokoto study, which was conducted in an urban tertiary health facility, our study was undertaken in a primary healthcare setting that provides more affordable, accessible, patient-centered, and potentially trauma-informed care. Primary healthcare also facilitates earlier patient contact and is generally less affected by the high patient volumes, administrative complexity, and logistical constraints that often contribute to delays in urban tertiary settings; thus, more pregnant women, including trauma-exposed parturients, are seen in the primary care setting. These findings underscore the considerable burden of intimate partner violence (IPV) and its adverse consequences in primary care settings, highlighting the importance of implementing context-specific screening and evidence-based interventions within primary healthcare to improve early identification and support for affected women.

The high prevalence of self-reported psychological distress among women exposed to IPV was reflected in the predominance of low self-esteem and sleep disturbance, reported by 77.1% and 67.3% of participants, respectively. These findings are consistent with previous reports demonstrating the substantial psychological burden associated with IPV exposure during pregnancy [8,10,12,14]. Furthermore, nearly one-third of IPV-exposed women reported suicidal ideation, a finding that aligns with previous studies demonstrating increased risk of severe psychological distress among women experiencing IPV [3,6]. The high prevalence of suicidal ideation among IPV-exposed women underscores the importance of adhering to the WHO ethical and safety recommendations for research on violence against women, which emphasize prioritizing participants’ safety, providing referrals to available local services and sources of support, protecting confidentiality, and ensuring appropriate training and support for research team members while maintaining methodological rigor [43]. Although these symptoms were self-reported rather than clinically diagnosed, they nevertheless indicate significant psychological vulnerability and reinforce the need to integrate trauma-informed mental health screening and assessment into routine antenatal care in primary healthcare settings for women experiencing IPV, preferably using multi-item validated instruments, such as the Rosenberg Self-Esteem Scale, which provides more robust measurement and facilitates comparison with similar studies [44,45].

Our finding of IPV-exposed women in pregnancy being more likely to initiate ANC early (≤12 weeks’ gestation) is consistent with the findings of Pagnini and Reichman, who reported that women who experienced domestic violence or abuse had elevated odds of early entry into prenatal care [25]. This observation is at odds with studies reporting that IPV exposure in pregnancy is associated with delayed initiation of prenatal care [22,46,47]. One possible explanation is that women experiencing IPV may perceive themselves or their pregnancies to be at greater risk and therefore seek antenatal care earlier because of concern for their own health and that of the fetus. It is also possible that differences in the setting or gestational timing of interviewer-administered IPV disclosure influenced participants’ willingness to disclose IPV, potentially introducing differential bias. Early ANC attendance, however, increases the opportunities for healthcare providers to identify IPV and provide necessary support and referral, particularly in the primary care setting.

Despite the high burden of self-reported psychological distress, no significant association was observed between psychological distress and delayed initiation of antenatal care. Similarly, exposure to IPV was not associated with delayed ANC attendance. These findings suggest that, in this population, neither self-reported psychological distress nor IPV exposure independently influenced the timing of ANC initiation. Many women experiencing IPV were already engaged with the primary healthcare system, presenting valuable opportunities for intervention during routine antenatal visits. This distinction is important because it shifts the focus from simply improving access to care toward maximizing the quality and scope of care delivered once women enter the primary care system.

Our findings have important implications for primary care practice, particularly in low-resource primary care settings where primary health centers are often the first—and sometimes only—point of contact for pregnant women. The high prevalence of IPV and accompanying psychological distress observed in this study supports the integration of routine IPV screening into ANC services. Such screening should extend beyond identifying IPV alone to include brief, trauma-informed psychological assessment and clearly defined referral pathways for women requiring mental health, social, and legal support. Integrating IPV screening into ANC reflects a multilevel intervention that addresses individual needs while strengthening connections between healthcare services and broader community support systems. By leveraging existing ANC encounters, primary care providers can play a pivotal role in identifying vulnerable women, facilitating timely assessment, referral, and psychological support, and potentially interrupting cycles of violence and poor maternal outcomes.

### 4.1. Implications for Future Studies

Future studies should adopt longitudinal or prospective designs to better establish the temporal pathways linking IPV exposure and ANC initiation. Such designs would allow researchers to examine whether psychological symptoms mediate or modify the relationship between IPV and maternal health service utilization. Given the high prevalence of multiple self-reported psychological distress indicators, future studies should assess composite mental health outcomes using validated multi-item measures for depression, anxiety, sleep disorders, and suicidality. Qualitative and mixed-methods research may also be warranted to explore why IPV-exposed women initiate ANC earlier, particularly considering conventional assumptions that IPV acts as a barrier to care. Understanding women’s lived experiences, coping strategies, and motivations for early care-seeking will provide critical contextual insights.

### 4.2. Limitations

Our study is a cross-sectional design, which limits causal inference. Although IPV exposure was associated with earlier ANC initiation, the findings may be due to residual confounding, selection bias, and the cross-sectional nature of the study. Also, our study relied on self-reported binary measures to assess IPV exposure and psychological distress indicators rather than validated multi-item instruments. This may have limited the depth and precision of assessment, particularly for sensitive experiences such as sleep disturbance and suicidal ideation, and may limit comparability with studies using validated measures. Finally, the study was conducted in a single primary health care setting, which may limit the generalizability of findings to other contexts, particularly tertiary facilities or rural areas with different care-seeking patterns and IPV dynamics.

## 5. Conclusions

Women reporting IPV during pregnancy also reported psychological distress indicators, including low self-esteem, sleep disturbance, and suicidal ideation, and may seek care early. These findings support integrating trauma-informed IPV identification, psychological assessment, and referral into routine antenatal care within primary healthcare settings.

## Figures and Tables

**Figure 1:**
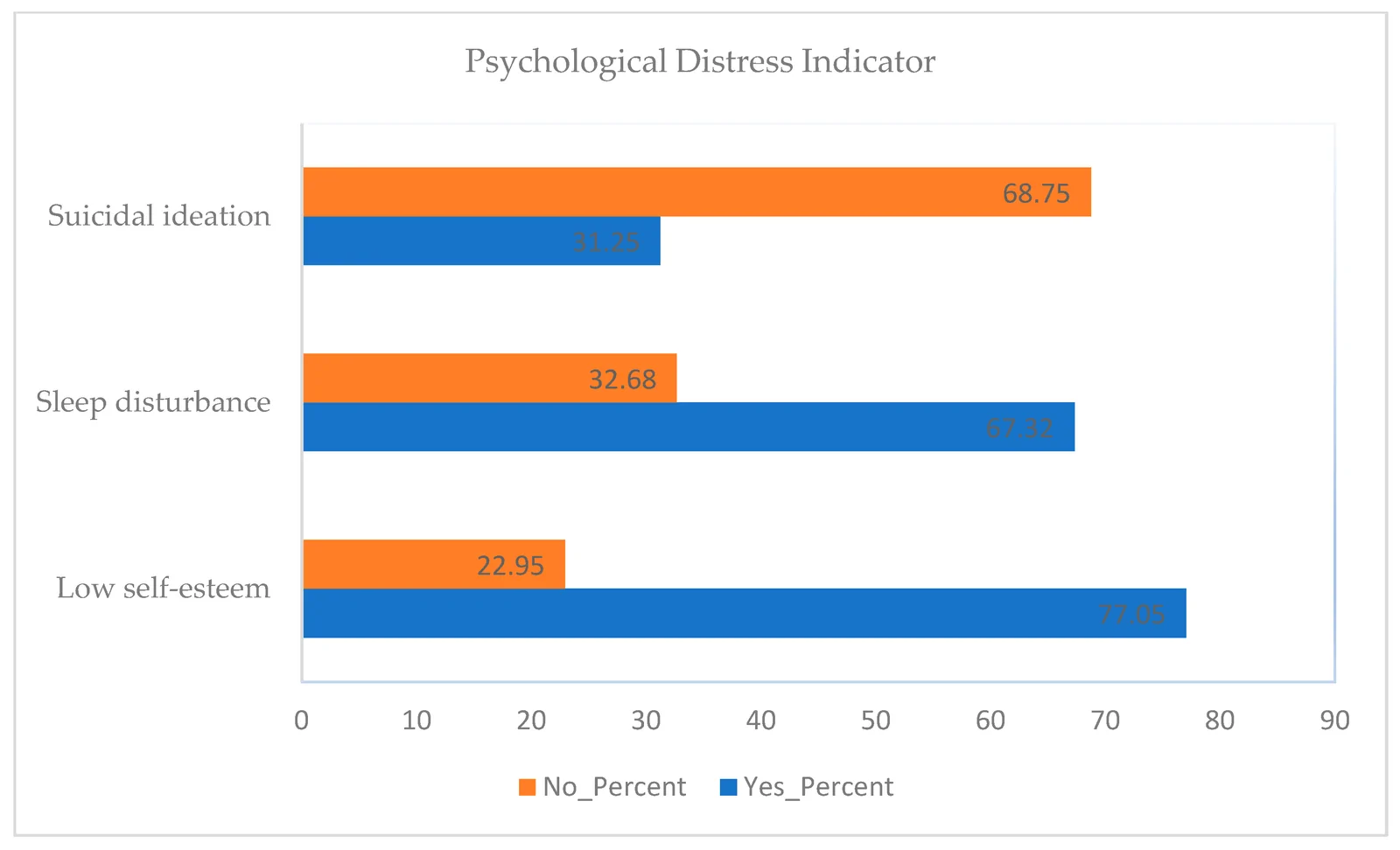
Self-reported psychological distress among IPV-exposed women.

**Table 1:** Sample characteristics by IPV exposure.

Variable	Exposure to IPV in Index Pregnancy		Total (*n* = 463)	*p* Value
	No IPV= *n* (%)	Yes IPV= *n* (%)		
	145 (31.3)	318 (68.7)		
**Maternal Age (yrs)**				0.712
<20	3(2.07)	6 (1.89)	9 (1.94)	
20–29	62(42.76)	145(45.60)	207(44.71)	
30–39	41(28.28)	97(30.50)	138(29.81)	
≥40	39(26.90)	70(22.01)	109(23.54)	
**Children Alive** Mean (SD)	2.88(1.76)	3.19(1.75)	3.1(1.76)	0.044 *
**Parity** Mean (SD)	3.51(2.06)	3.96(1.98)	3.82(2.01)	0.015 *
**Maternal Education**	0.004			
Primary	24 (16.55)	86(27.04)	110(23.76)	
Secondary	36(24.83)	45(14.15)	81(17.49)	
Tertiary	85(58.62)	187(58.75)	272(58.75)	
**Maternal Occupation**	0.011			
Farmer	9(6.21)	37(11.64)	46(9.94)	
Artisan	80(55.17)	125(39.31)	205(44.28)	
Civil servants	54(37.24)	149(46.86)	203(43.84)	
Others	2(1.38)	7(2.20)	9(1.94)	
**Antenatal Booking (GW)**	<0.001			
≤12 weeks	38(26.21)	155(48.74)	193(41.68)	
>12 weeks	107(73.79)	163(51.26)	270(58.32)	

* Bivariate analysis by *t*-test; others: chi-square; SD: standard deviation; GW: gestational weeks.

**Table 2:** Association between IPV and *delayed ANC initiation* (Model 1).

Late ANC Initiation	aOR (95% CI)	*p* Value
**IPV in index pregnancy**	0.37 (0.19–0.69)	0.002
**Maternal Age**		
**20–29**	1.70(0.369–7.83)	0.496
**30–39**	1.27(0.27–6.01)	0.767
≥40	0.82(0.16–3.97)	0.808
**Parity**	1.10(0.87–1.39)	0.411
**Children Alive**	1.07(0.82–1.40)	0.586
**Maternal Education**		
Secondary	2.10(1.08–4.08)	0.028
Tertiary	2.52(1.39–4.55)	0.002
**Maternal Occupation**		
Farmer	0.94(0.39–2.24)	0.884
Artisan	0.65(0.38–1.10)	0.108
**Partner Education**		
Secondary	0.92(0.43–1.97)	0.838
Tertiary	1.04(0.53–2.03)	0.906
**Partner Occupation**		
Farmer	0.22(0.09–0.51)	<0.001
Artisan	0.31(0.19–0.50)	<0.001
**Partner job insecurity**	0.74(0.42–1.35)	0.336
**Partner substance abuse**	0.81(0.26–2.53)	0.716
**Low social support**	0.68(0.33–1.42)	0.303

Unadjusted OR 0.37(0.24–0.57) *p* < 0.001. Reference category: age < 20; education maternal/partner: primary; occupation maternal/partner: civil servant.

**Table 3:** Association between psychological distress indicators and ANC timing among IPV-exposed women (Model 2).

ANC Booking Timing	aOR (95% CI) *p* Value		aOR (95% CI) *p* Value		aOR (95% CI) *p* Value	
	Low Self-Esteem 1.01(0.53–1.92)	0.982	Sleep Disturbance 0.83 (0.47–1.47)	0.527	Suicidal Ideation 0.91 (0.48–1.75)	0.787
**Maternal age**						
**20–29**	3.77(0.35–40.18)	0.272	4.11(0.41–41.48)	0.230	4.27(0.42–43.01)	0.218
**30–39**	2.99(0.26–33.56)	0.373	3.27(0.31–34.55)	0.324	3.37(0.31–35.75)	0.313
**≥40**	1.77(0.16–20.29)	0.644	1.95(0.18–21.18)	0.580	2.00(0.18–21.75)	0.569
**Parity**	1.09(0.81–1.48)	0.542	1.11(0.822–1.51)	0.484	1.09(0.81–1.48)	0.536
**Children Alive**	1.22(0.87–1.72)	0.249	1.19(0.85–1.69)	0.306	1.21(0.87–1.71)	0.253
**Maternal Education**						
Secondary	3.65(1.55–8.59)	0.003	3.57(1.52–8.39)	0.003	3.58 (1.52–8.40)	0.003
Tertiary	3.42(1.66–7.04)	0.001	3.44(1.68–7.07)	0.001	3.41(1.66–7.01)	0.001
**Maternal Occupation**						
Farmer	0.58(0.20–1.63)	0.302	0.59(0.21–1.66)	0.314	0.58 (0.21–1.64)	0.306
Artisan	0.55(0.29–1.06)	0.075	0.57 (0.29–1.08)	0.086	0.55(0.29–1.07)	0.079
**Partner Education**						
Secondary	0.55(0.21–1.47)	0.237	0.55(0.21–1.47)	0.234	0.54(0.19–1.46)	0.225
Tertiary	0.77(0.34–1.74)	0.534	0.78(0.35–1.74)	0.542	0.76(0.34–1.71)	0.510
**Partner Occupation**						
Farmer	0.36(0.13–1.04)	0.059	0.36(0.13–1.04)	0.058	0.36(0.13–1.03)	0.056
Artisan	0.34(0.18–0.62)	<0.001	0.34(0.19–0.62)	<0.001	0.33(0.18–0.61)	<0.001
**Partner job insecurity**	0.73(0.39–1.36)	0.327	0.73(0.39–1.35)	0.318	0.74(0.39–1.37)	0.339
**Substance abuse**	0.74(0.22–2.46)	0.620	0.70(0.21–2.36)	0.571	0.73(0.22–2.43)	0.604
**Low social support**	0.71(0.33–1.56)	0.403	0.73(0.34–1.60)	0.441	0.73(0.34–1.58)	0.426

Reference category: age < 20; education maternal/partner: primary; occupation maternal/partner: civil servant.

## Data Availability

The data presented in this study are available upon reasonable request from the corresponding author due to privacy and ethical restrictions.
